# Implementing an Assessment Clinic in a Residential PTSD Program

**DOI:** 10.3390/bs4030243

**Published:** 2014-08-06

**Authors:** Joan McDowell, Eliza McManus, Jessica L. Rodriguez

**Affiliations:** 1 Battle Creek Veterans Affairs Medical Center, 5600 Armstrong Road, Battle Creek, MI 49037, USA; E-Mail: jessica.rodriguez5@va.gov; 2 Western Michigan University, 1903 West Michigan Avenue, Kalamazoo, MI 49008, USA; E-Mail: eliza.s.mcmanus@wmich.edu

**Keywords:** evidence-based assessment, implementation, PTSD residential treatment program

## Abstract

Creating useful treatment plans can help improve services to consumers of mental health services. As more evidence-based practices are implemented, deciding what treatment, at what time, for whom becomes an important factor in facilitating positive outcomes. Readiness for trauma-focused treatments for Posttraumatic Stress Disorder (PTSD) such as Cognitive Processing Therapy or Prolonged Exposure Therapy may influence whether an individual can successfully complete either protocol. In addition, components of adjunctive therapies such as Acceptance and Commitment Therapy or Dialectical Behavior Therapy may be useful in moving a particular patient toward readiness and successful completion of treatment. Psychological assessment adds valuable data to inform these types of treatment decisions. This paper describes the implementation of a psychological assessment clinic in a residential PTSD treatment setting. Barriers to implementation, use of the data, and Veterans’ reactions to the feedback provided to them are included.

## 1. Introduction

Although a stress response syndrome as a result of severe traumatic experiences has been around since the beginning of time, recognition of a set of symptoms defined as a disorder in the psychiatric diagnostic manual three decades ago sparked an explosion of research surrounding Posttraumatic Stress Disorder (PTSD). We know that exposure to trauma and resulting PTSD are relatively high among the general population [1], as well as among military personnel exposed to combat [2]. With the terrorist attacks on 11 September 2001 and the ensuing war in the Middle East, PTSD has become a common term, often seen in mainstream media, newscasts, and even in fictional entertainment. In addition, more attention is being paid to treatment for PTSD, especially with increased interest in complex PTSD or Disorders of Extreme Stress, Not Otherwise Specified (DESNOS) [3,4,5]. This has resulted in a number of assessment instruments and treatment methods from which to choose.

Meeting professional standards and best practice recommendations for evidence-based care involves having accurate diagnoses and case conceptualizations to guide treatment, as well as monitoring progress in treatment to inform and adapt as necessary [2,6]. This includes the use of validated assessment instruments at intake as well as re-administering measures periodically in order to evaluate effectiveness of treatment. Treatment outcomes may be affected by decisions made early in a patient’s treatment. A thorough assessment is likely to improve decision making by providing detailed information regarding PTSD symptoms and comorbid conditions, as well as other factors unique to the patient such as coping mechanisms and personality functioning. Secondly, assessment can assist with accurate diagnosis of PTSD since there may be secondary gain factors that impact a patient’s description of symptoms. Finally, routine assessment can provide data to guide program development and improve treatment. This information can be used for improvement projects, such as enhancing staff training or the addition of adjunct treatments to supplement existing offerings in the treatment program.

In addition to the benefits listed above, research suggests that there may be secondary benefits to integrating assessment feedback into therapy sessions for patients. One study showed that patients who had been involved with regular assessment feedback sessions during their treatment felt they had more involvement with treatment decisions, had more respect and dignity, and they also were more likely to recommend the treatment facility to others [7]. However, gathering information and providing feedback to patients can prove challenging. The literature on implementation science suggests that most implementation projects are difficult, frequently fail, and take months or years of effort [8,9]. Finding ways to streamline this process can improve the availability of information to use in the treatment of PTSD.

This article describes the process of implementing an assessment clinic at the Battle Creek Veterans Affairs (BCVA) PTSD-Residential Rehabilitation Treatment Program (PTSD-RRTP) beginning with the selection of assessment instruments, describing the logistics of integrating the clinic into the treatment program, and finally discussing future directions for this type of assessment clinic. The need for such a clinic was supported by increased recognition of the acuity and complexity of the Veterans entering the program. The assessment clinic was designed to assist with treatment planning for the types of complex cases often admitted to residential treatment programs. The primary objective for this project was to make a formal psychological assessment available for each admitted patient that would be useful for diagnosis, treatment planning, and also as the basis for an intervention when necessary. Describing the steps involved in overcoming problems, such as financial or logistical obstacles, may help others in anticipating barriers to implementing assessment clinics in their own settings.

## 2. Implementation Science

Implementation refers to the process of putting new systems into effect [10] and has been a fundamental part of quality improvement activities in industrial settings for many years. More recently, there have been increasing efforts to utilize implementation science principles in health care systems [9,11]. For example, the VA Quality Enhancement Research Initiative seeks to provide a framework for dissemination and implementation of best practices within its network of medical centers [12]. Utilizing an implementation framework provides a foundation for systematic change that is more likely to meet with success and sustainability. Frameworks provide a set of procedures that focus a project on understanding contextual factors at the patient, provider, and system levels that may affect implementation efforts; help in developing a plan for implementation; and facilitate monitoring of outcomes for sustainability.

One such framework that has been useful for implementation of new processes within health care settings is the FOCUS-PDSA model (Find, Organize, Clarify, Understand, Select—Plan, Do, Study, Act) [13]. The PDSA portion of the framework is credited to Edward Deming who was an early pioneer in the quality improvement movement for industrial processes [14]. The PDSA method has become widely used in the field of healthcare for quality improvement as well. It is based on the scientific method (hypothesize, test, analyze, revise), which is familiar to scientist-practitioner psychologists. The FOCUS portion of the framework was added later through work at the Hospital Corporation of America [13]. FOCUS was added before the PDSA cycle with the intention of gathering the information necessary to facilitate the process of adaptation. A review article that sought to find consensus on best practices for implementation of evidence-based psychological treatments reported that in-depth assessment of needs and management of barriers was common across programs and likely leads to better outcomes [11]. The FOCUS portion of the framework described in this article provided the opportunity to fully explore aspects of the system that may lead to failure prior to implementing any of the planned changes. There are a number of other frameworks [see Reference 9 for a review], but the FOCUS-PDSA framework will be the model used throughout the remainder of this text to describe how the current project was implemented.

## 3. Step One: *Find* a Process to Improve

The psychologists in the PTSD-RRTP had struggled to maintain a consistent psychological assessment program after the psychology technician’s retirement left a vacancy that was not back-filled. Additional backfilled psychology positions brought in new employees and increased the interest in revitalizing the assessment clinic. Early in this process, one clinician became a champion for the project. This psychologist eventually elected to serve as the implementation facilitator and ongoing manager for sustainability of the assessment clinic. In addition to having an interest in the project and some knowledge of the processes under consideration, an implementation facilitator should also be credible, persuasive, engaging, and persistent.

## 4. Step Two: *Organize* a Team

In any system, thoughtfully considering who the stakeholders may be can provide information about who to enlist for the implementation project. Not only would this include the people directly involved in the clinic, but may also include additional personnel who are responsible for ancillary duties such as scheduling, or personnel who may be instrumental in securing the necessary resources.

The champion at BCVA enlisted support from the PTSD-RRTP manager as well as other influential peers to work in a small committee to discuss the possibility of administering a thorough, consistent psychological assessment for every patient admitted to the program. Engaging a member of management early in the process helped assure that there would be support available for the necessary resources as well as another source of social influence. Peer input helped to assure buy-in from frontline clinicians, as well as assisting with identifying catalysts and barriers from an array of perspectives.

## 5. Step Three: *Clarify* Current Knowledge of the Process

The committee identified four primary goals. The first goal is to gather and synthesize information useful for determining a preliminary diagnostic picture. The data obtained in the assessment can help to inform the primary therapist’s diagnostic decisions by supplementing the information obtained during an intake interview. In addition to the assessment clinic, each patient meets with a primary therapist for an interview to integrate all of the information and fill in any blanks to obtain as complete a picture of the patient’s functioning as possible. Identifying preliminary diagnoses enables treatment providers to make faster and better decisions to determine the best course of treatment for the Veteran or to make appropriate treatment referrals. For example, if psychological assessment indicates that presenting concerns are consistent with a severe major depressive disorder that would complicate trauma-focused treatment, efforts could be made to address this problem.

The second identified goal of the assessment clinic is to gather relevant information that can be used to tailor an individualized treatment plan. Most residential treatment programs have a variety of evidence-based psychotherapy and psychoeducation groups designed to address a number of presenting symptoms, concerns, and goals. The vast number of treatment options can be overwhelming for Veterans. While primary therapists provide guidance to patients about which groups and classes to attend, the assessment results can help bolster the therapists’ treatment recommendations. After receiving the completed assessment results, the Veteran’s primary therapist could meet with the Veteran to discuss the findings and provide education about groups or activities that may be particularly beneficial. For example, a therapist may refer a Veteran to specialty treatment for insomnia after reviewing preliminary measures of insomnia severity and discussing the Veteran’s sleep difficulties during the assessment feedback session. In this way, the patient gets a tailored approach early in residential treatment that optimizes treatment gains.

Developing a treatment plan includes considering whether a Veteran is likely to benefit from residential treatment for PTSD at a specific point in time, and more specifically, from trauma-focused therapies that are the basis for treatment of PTSD. This is a significant consideration given the importance of utilization of resources in a specialized residential treatment program. Information garnered in an assessment clinic can help inform decisions about appropriateness for treatment. For example, while research regarding prognostic indicators for treatment is at best mixed, some studies have found a relationship between treatment dropout/outcome and depression, anger, hyperarousal, guilt, and social support (e.g., [15,16,17,18]). Measures that provide information regarding these domains can be immensely useful in identifying patients that may require additional support or pre-trauma-focused interventions. Validity measures may also be helpful in identifying individuals who could benefit from other therapeutic interventions prior to beginning trauma-focused treatment. It is well-established that some populations, such as combat Veterans, frequently exaggerate symptoms [19], which would suggest that initial interventions aimed at improving expectancies for treatment or focused on reducing global distress may enhance treatment outcomes. Although the assessment process may help identify individuals with these types of characteristics, certainly treatment recommendations are never made based on one assessment finding. Data from the assessment results should be integrated with information from other sources to facilitate treatment recommendations.

The third goal identified for the assessment clinic is to promote Veteran awareness and engagement in treatment. Primary therapists can use a feedback session as a therapeutic intervention to discuss change (or lack of change), engagement in treatment, additional steps that may be necessary before engaging in trauma-focused therapy, or to enhance motivation for additional work such as increasing exposure opportunities. In this way, the Veteran is provided objective feedback at each stage of treatment and is able to make informed decisions about treatment. Providing therapeutic feedback to the Veteran at the beginning, mid-point, and end of residential treatment promotes awareness and personal responsibility for treatment [20].

The final goal of the assessment clinic is to gather information that can be used for continued performance evaluation of the PTSD-RRTP and other research oriented activities. Collecting outcome measures in a systematic manner allows psychology staff to evaluate and monitor the efficacy of the treatment program, which can provide support for additional staff trainings or adjunctive treatment opportunities within the residential program as well as a longitudinal data set to help answer research questions.

Identifying and defining the goals for the process under study is an extremely important step that helps provide direction for the rest of the process. For example, there may be some settings with little interest in aggregating the data for evaluation and research activities. The main focus may be on each individual patient’s care and only used for that purpose. If this were the case, the project becomes somewhat smaller in scope without the need for a database and personnel to input and interpret the data.

Clarifying the process also involved reviewing how assessments had been conducted in the past and gathering input from clinicians concerning how they had observed similar processes in other settings. For example, an assessment clinic had previously been implemented at the Battle Creek VAMC utilizing a psychology technician. Another program used for comparison was implemented at the C.W. Bill Young VA Medical Center (formerly the Bay Pines VA Healthcare System). This step involved examining the assessment process with respect to how well it accomplished the identified goals and met benchmark standards exhibited by these comparison programs.

## 6. Step Four: *Understand* Causes of Variation

**Figure 1:**
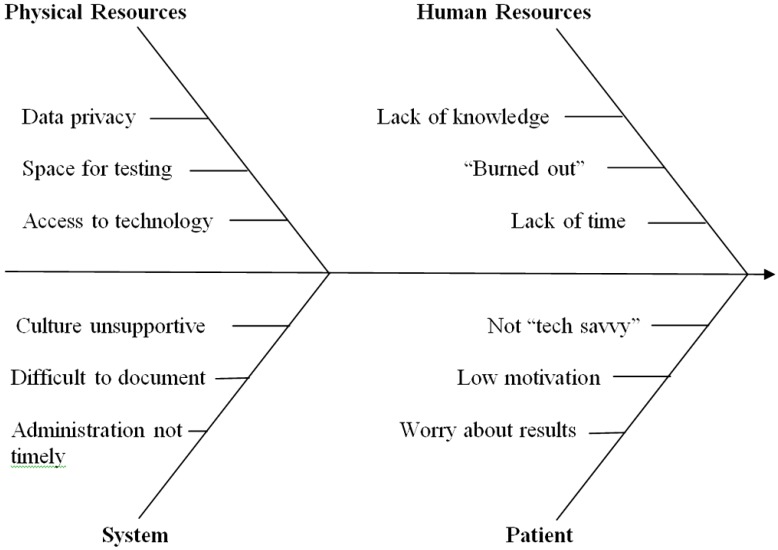
Factors affecting implementation of an assessment clinic.

This is a critical step in the implementation process because this is where the team identifies both barriers and catalysts for the process under study. Psychological assessment had been established in the PTSD-RRTP; however, it was not always consistent, useful, or meeting the goals defined in the previous stage. A number of problem areas were identified as targets for intervention. The fishbone diagram in Figure 1 below illustrates the areas that were identified.

## 7. Step Five: *Select* the Process Improvements

Matching process improvements to each of the problem areas identified in the preceding step was the target for this stage. Due to the complexity of the issues involved, it was necessary to have a multistep plan that would begin interventions in a number of areas simultaneously. For example, interventions were necessary in the physical environment to procure technological and physical space resources for the project at the same time that process improvements were identified to address system issues that did not support the assessment clinic. Particular attention was paid to staff issues since the time investment was a major barrier to the success of the project. Finally, it was also important to address patient discomfort with the testing process in order to meet the goals specified above. The next step created detailed plans for implementing each of the identified improvements and is described in the following section. This concluded the first half of the implementation effort and the project moved into the PDSA cycle to complete the process.

## 8. Step Six: *Plan* a Change

This involved front-line treatment providers from a variety of mental health disciplines as well as the program manager. The small committee discussed the needs of the clinic and brainstormed possible solutions on a regular basis throughout the implementation process. The results of these meetings were discussed periodically in full treatment team meetings for more input. The meetings also served the purpose of overcoming challenges with staff regarding buy-in, workload issues, and training and education. The plans addressing each of the issues noted in the fishbone diagram are described in more detail below.

### 8.1. Selection of Instruments

A priority of the plan was to select instruments for the battery that would be routinely administered. There is no one superior measure for assessment of PTSD; selection depends on goals and objectives as well as constraints of the system that will be utilizing the measure [21]. Goals and objectives (e.g., speed *vs.* accuracy) as well as base rates may vary from setting to setting. Defining a specific set of instruments is not the basis for this article. Rather, we discuss the selection process that we utilized and the conclusions drawn to help other clinicians and researchers through the decision making process. Consistent with evidence-based practice guidelines, the decision-making process sought to integrate information from the best available research, clinical expertise, and patient characteristics [6].

Feedback from the treatment team staff suggested that the assessment clinic should be heavily oriented towards gathering information that would be clinically useful for therapists and Veterans. Therefore, each instrument was carefully examined for how the data would be used in our specific program. The committee, with input from mental health staff, selected measures that were relevant to the PTSD population served and had evidence supporting their reliability and validity. As noted above, patients admitted to residential treatment programs typically have a constellation of complex problems. Given the specialized nature of the PTSD-RRTP, mental health staff were interested in measures that covered core PTSD symptoms (*i.e.*, reexperiencing, avoidance, and increased arousal), as well as other problems that may be associated with PTSD such as substance misuse, anxiety sensitivity, and depression.

Criteria were identified for selection of instruments, which served to narrow down the possibilities. Time to administer and score, cost, empirical support, and sensitivity to change were primary selection variables. Although the use of a structured clinical interview would undoubtedly provide useful information (and was strongly considered early in the process), the argument for efficiency took precedence. Other settings with different goals or resources may come to a different conclusion for their own assessment clinics. Self-report instruments became the basis for the battery, with an emphasis on instruments that could be administered and scored via computer. Self-report scales have obvious limitations, therefore, the selection of instruments focused on measures that had substantial evidence demonstrating reliability and validity. In addition, the inclusion of measures that included validity indices helped to ensure that accurate data would be collected.

Secondly, instruments already available to the VA through preexisting contracts or which are available in the public domain were a primary target for selection. This not only served to speed the process of implementation (since time was not dedicated to procurement issues), but also increased acceptability for administrators by reducing costs associated with this project. For example, it is estimated that with roughly 300 unique admissions each year the medical center saved approximately $780 annually for a single instrument by using an already available measure (anger content scale of the MMPI-2) [22] rather than purchasing another instrument. Multiplying this by the ten instruments in the battery results in a significant savings. The instruments accessible to specific clinics or medical centers may vary, but choosing to begin with instruments that are already available can prevent a project from becoming bogged down. Addition of other measures that may not have been available at the time of the initial implementation is much easier on a piecemeal basis after the program is established.

Because Veterans would be assessed at multiple time points during residential treatment, it was also important to include a number of instruments that were sensitive to change. Measures of substance use, mood, anxiety, and sleep difficulty as well as a PTSD symptom inventory were selected as repeated measures to monitor treatment progress and to help in planning treatment changes while the Veteran is still engaged in residential programming. For example, a Veteran whose scores indicate a decrease in overall posttraumatic stress disorder symptoms but continued avoidance symptoms may be encouraged during their assessment feedback session to increase exposure-related activities. In this manner, therapists can target specific problem areas for intervention. During the feedback session this goal may be added to the Veteran’s treatment plan to target during the remainder of their residential stay or as a recommended target when the Veteran returns to outpatient care.

The Minnesota Multiphasic Personality Inventory—Second Edition (MMPI-2) [23] was also selected for the assessment clinic, as it is a commonly used and extensively researched measure of personality functioning and psychopathology that provides a wealth of information. This measure is useful in identifying the adjustment level and characteristic traits and behaviors of the individual, which may aid with identifying appropriate treatment recommendations. While not specifically a measure of PTSD, at current review nearly 300 peer-reviewed articles are listed on Psych Info and over 7000 citations are listed on Google Scholar examining the relationship between the MMPI-2 and PTSD symptomology. Numerous articles have been published regarding the common symptom profiles for individuals with PTSD and specific PTSD scales within the MMPI (e.g., [24,25,26,27,28]). Another reason for the addition of the MMPI-2 was the presence of validity scales. One of the difficulties of relying primarily on self-report measures is the lack of information regarding how forthright an individual is while completing the measures. By adding an instrument with many validity indicators, providers can examine the test taking approach of each examinee before proceeding with more in depth interpretation [29]. For example, inconsistent responding, an acquiescence bias, defensiveness, underreporting, exaggeration of symptoms, and many other response styles can be detected with the use of the MMPI-2. This allows providers the opportunity to examine the accuracy of responses and have greater confidence in the ability or inability to further interpret and use assessment information.

Finally, some measures were selected based on their use in other residential treatment programs at the Battle Creek VAMC in an effort to provide standard measures across time in cases where Veterans are transferred from one treatment program to another (e.g., substance abuse to PTSD or vice versa). One example is the inclusion of the Brief Addiction Monitor (BAM) [30], which provides information regarding substance use in the past 30 days, as well as risk and protective factors for substance use.

Table 1 lists the instruments that were selected for the PTSD-RRTP assessment clinic. For a more thorough review of assessment instruments for PTSD, see [31].

**Table 1:** PTSD-RRTP Assessment Battery.

Domain	Instrument	Psychometric Studies
Anxiety	ASI-III	[32]
Cognitive Flexibility	CFS	[33]
Depression	BDI-II	[34]
Functioning	BASIS-24	[35]
Insomnia	ISI	[36]
Personality Functioning	MMPI-2	[23,25]
Posttraumatic Growth	PTGI-SF	[37]
PTSD	PCL-C	[38]
Substance Abuse	BAM	[30]
Traumatic Life Events	LEC	[39]

Note: ASI-III (Anxiety Sensitivity Index-Third Edition); CFS (Cognitive Flexibility Scale); BDI-II (Beck Depression Inventory—Second Edition); BASIS-24 (Behavior and Symptom Identification Scale); ISI (Insomnia Severity Index); MMPI-2 (Minnesota Multiphasic Personality Inventory—Second Edition); PTGI-SF (Posttraumatic Growth Inventory—Short Form); PCL-C (Posttraumatic Checklist—Civilian Version); BAM (Brief Addiction Monitor); and LEC (Life Events Checklist).

### 8.2. Logistical Considerations

#### 8.2.1. Space Issues

The assessment clinic needed a dedicated space for test administration. While space limitations at the time of implementation made it impossible to have a room that was specific to the assessment clinic, a multipurpose room (treatment team/group therapy room) was identified that could be shared. The room was reserved for three-hour blocks of time on the same days each week for the assessment clinic. The room already contained four computers and a long table with enough workspace for Veterans to complete paper and pencil measures, thereby limiting the initial investment in resources. However, the number of computers limited the number of Veterans who could be tested each day so the assessment clinic was scheduled for two times each week. Within their first week in residential treatment, patients receive an appointment with instructions to present to the appropriate room for psychological testing. Veterans are allowed short breaks and most complete initial testing in less than two hours.

#### 8.2.2. Timing of Administrations

To capture treatment gains, the self-report measures are re-administered at the time that a Veteran changes from one phase of treatment to another (*i.e.*, coping skills to trauma-focused therapy) and prior to discharge from the residential program. The additional feedback sessions are an opportunity for the therapist to highlight any changes in symptoms and to focus on areas that continue to need improvement. This can enable the Veteran to direct efforts towards additional areas while they are still in residential treatment, identify any reduction in symptoms over the course of treatment, and select further improvements that they may wish to work on in outpatient therapy.

#### 8.2.3. Time Issues

The time investment for clinicians with already full workloads was identified as one of the biggest barriers to implementing this project. Not only is there a need for a clinician to manage the program on an ongoing basis, but staff is necessary for continued administration, scoring, and interpretation of the measures. The implementation facilitator/manager is responsible for monitoring the scheduling of assessment appointments to assure that patients receive testing at the appropriate time points. Secondly, this provider is responsible for supervising the administration, scoring, and interpretation of the assessment data.

In addition to the time commitment of managing the clinic, there were concerns about the time necessary to administer, score, and interpret the tests, and to provide feedback on the results to each Veteran. Several strategies were implemented to enhance efficiency and reduce additional burden on the clinicians working in the PTSD-RRTP.

One important method of increasing efficiency was the development of a template to streamline assessment results. Using a standardized template shortens the amount of time needed to write the assessment results, both decreasing the time burden and allowing for more timely feedback to the Veteran. Because the information becomes part of the medical record that Veterans can access, the template is written in a manner that is logical and clear so the results can be easily understood. This has the added benefit of reducing the time invested by primary therapists. The consistent format speeds understanding and the simplified results are easily understood by therapists from all disciplines, regardless of their level of experience with psychological assessment. Since the template includes scoring parameters and standardized interpretations, a further benefit is consistency across patients and reduced scoring errors.

Another important strategy for reducing burden was the use of psychology students to proctor, score, and interpret the assessments. The treatment team initially considered the use of a bachelor’s level psychology technician for administration and scoring. However, in early committee meetings it was determined that this option was not available at the medical center at the time of implementation. Some larger settings may consider hiring a psychology technician to administer psychological testing or using existing resources within the organization. Another alternative for smaller organizations may be to contract for services outside the system.

Since none of these options were available at the time, master’s level psychology practicum students from local universities were recruited to function as test administrators. This serves a dual purpose by limiting burden on existing staff members as well as fulfilling a secondary mission for BCVA to provide valuable training opportunities for students.

Psychology students seeking to enhance their experiences are available in most areas. Universities do not need to be in the local area since students will drive quite a distance for a placement in a mental health setting. Not only do students want to expand their skills, improving one’s *curriculum vitae* increases the chances for placement at internship and postdoctoral positions. Therefore, recruitment involves identifying nearby universities and contacting each training director to offer information about the experience that is available. Maintaining these relationships provides a steady flow of students eager for the opportunity to work in the clinic. One *caveat*, however, is to ensure that students receive proper supervision as a key part of the process.

In our assessment clinic, the practicum students are trained to adhere to the assessment protocol. Students learn to administer a number of measures, score and interpret the measures, and write assessment reports under the supervision of psychology staff. As noted above, a template is utilized to promote consistency in reporting results and to minimize the time burden for staff and trainees. This also expedites training each new group of practicum students as they take over for the outgoing students.

#### 8.2.4. Staff Issues

The treatment team believed that the assessment feedback would have the most therapeutic benefit if it were to come from the Veteran’s primary therapist in the context of a therapy session. Each Veteran is assigned a primary therapist at admission who is instrumental in managing the patient’s treatment. The patient meets with the primary therapist at regular intervals. In addition to providing therapeutic interventions that build on the group programming, the primary therapist serves as the contact point for navigating any difficulties that may arise during treatment (e.g., behavioral issues, nonattendance, lack of progress) and for reinforcing gains and helping the patient to continue to build on recovery. Therefore, the Veteran’s individual therapist is expected to integrate assessment feedback throughout treatment into the individual therapy sessions. It begins as primary therapists share the feedback and treatment recommendations with each Veteran within a week of receiving the assessment report. The feedback occurs during a regularly scheduled therapy session, thereby limiting any additional time commitment from staff. Given the emphasis on treatment planning, the need for primary therapists to provide feedback that was clear and easy to understand was emphasized.

Primary therapists are also encouraged to utilize the repeated assessment data to monitor progress. This provides opportunities to address situations in which there is little progress in treatment. Helping a patient confront avoidance or other secondary gain issues that maintain symptoms is facilitated by introducing the data that was reported by the patient. Or alternatively, repeated assessments can help patients build confidence by showing them objective measures of their gains. This can be extremely valuable information since many patients continue to focus on strong emotions they experience, while discounting other signs of progress.

In order to facilitate successful feedback sessions, training opportunities for treatment team members were provided at several points in the implementation process. Familiarity with the instruments was ensured by having periodic discussions and reviews during treatment team meetings. Additionally, since not all primary therapists are psychologists, training for mental health staff on methods of sharing feedback was provided, which is explained in more detail later in this article. The use of a simple template, described above, likewise helped with supplying information to clinicians that was well-received. The template provides information in a way that does not rely on sophisticated statistical knowledge to be useful.

To address the concerns regarding time constraints of individual providers and resistance to taking on additional work, the inception of the assessment clinic focused on providing information that is seen as helpful to the clinician while minimizing the time, effort, and burden on individual providers. To accomplish this task, psychology trainees were used to administer, score, and interpret the majority of the assessment cases, as described above. Additionally, specific providers with particular interest and knowledge in assessment were recruited to interpret overflow assessments. In our clinic, because of the measures selected (e.g., MMPI-2), it required that only psychologists and psychology students under the supervision of a psychologist interpret the results. Three psychologists elected to take on this role, interpreting and entering the assessment results in medical records for approximately two to three assessments per week. With the frequency of interpretation and use of the assessment template, results can often be interpreted in as little as 15 minutes. However, more complex assessment results may require a greater amount of time. Overall, these steps have effectively addressed staff concerns regarding a possible burden placed on them by the assessment process, while allowing them the benefit of receiving the assessment results. Although this procedure worked well for the current program, it is not a one size fits all process. For example, other sites may elect to have each provider complete the assessment reports on their individual clients, spreading the workload more evenly.

#### 8.2.5. Technology Issues

While the decision to use computer-assisted measures saves time for both the Veteran and technician, it is not without issues that needed to be considered when laying the foundation for the assessment clinic. The clinic required access to a number of computers for administration and a printer so that measures could be printed out for the appropriate staff for interpretation or feedback. Involvement of management was important for procuring the resources necessary in this area, especially as the clinic expanded into a dedicated workspace. The issue of privacy was important to address given that the computers were connected to the main server accessing all medical records. The implementation committee sought out VA computer support staff to ensure that computers could be placed in a “secure” desktop mode which disabled sensitive options such as the electronic medical record and VA e-mail programs, and which also automatically logged the Veteran out of the computer after testing was complete.

## 9. Step Seven: *Do* the Change

The changes identified during the planning stages were carried out in a prioritized fashion. For example, procuring the necessary technologies and selection of instruments had to proceed before any Veterans could be scheduled for testing. However, many of the planned changes were implemented more or less simultaneously as time and opportunity allowed.

Although the PDSA cycle suggests a linear process, this project (and likely many other complex projects) may have steps operating simultaneously. Consequently, changes were taking place as part of the *Do* stage even while additional *Plan* steps were still under consideration. For example, the treatment team meetings, while useful for gathering additional feedback on the proposed changes, also served as an educational forum for increasing team members’ knowledge of assessment literature and as a means of influencing the social system to create buy-in and support for the program. Continual attention to progress and refocusing priorities during the each stage helps ensure the project does not get stalled. Much of this is accomplished by moving fluidly back and forth between the *Plan, Do, Study*, and *Act* processes.

## 10. Step Eight: *Study* the Effects

Evaluation of the changes made during the *Do* step was conducted on a regular basis throughout the implementation. As noted above, the PDSA cycle may not operate in a completely linear fashion. Monitoring the number of Veterans who did not complete the clinic, soliciting feedback from primary therapists involved with the Veterans, taking note of issues (such as a shortage of computers), and soliciting feedback from the Veterans themselves provided information for continual improvement, which is the primary goal of the PDSA cycle.

## 11. Step Nine: *Act* on the Results

Studying the effects of the changes helped to identify challenges to the continued success of the assessment clinic as well as additional opportunities. Increased workload and time demands, patient aversion to the assessments, staff members’ lack of familiarity with the measures, monitoring the stages of the assessment process, timely provision of feedback, and training additional clinicians to monitor the assessment clinic were areas that required further efforts to address.

The amount of time taken to administer, score, and interpret the assessments is significant, especially when added on top of the taxing workload of many clinicians. The current model in which psychology practicum students were trained to proctor, score, and interpret assessments is also initially time consuming. However, it became apparent that it would save time in the long run as students became increasingly autonomous. Interestingly, as discussion between students increased related to the valuable training opportunities afforded in the assessment clinic, so too did our applications for practicum students. Historically, we have had one master’s level psychology practicum student working in the PTSD program each year. The assessment clinic began with this one student assisting. Within six months, several other students had requested appointments at the medical center. After a year, the number of practicum students involved in the process had risen to four through word of mouth. The old adage, “if you build it, they will come” seems very appropriate in this situation. Our students have an additional opportunity for a tiered supervision experience training new students in the assessment process. As the VA is the largest provider of psychology training in the United States [40], this model likely will fit within the structure of similar training sites.

Aversion to the assessment process, apparent with some Veterans, posed another barrier to implementation. While a minority of overall assessment cases, some Veterans expressed complaints regarding the length of the assessment, repetitiveness of some questions or measures, and concern regarding how the results would be used (e.g., whether they will be sent to the inpatient psychiatric unit based on their responses, whether they will lose their benefits or not get into treatment if the assessment shows they do not have PTSD). Informed consent is a standard part of the assessment process; however, it was found that spending 2–5 additional minutes with these Veterans to address their concerns was helpful. Reviewing information about confidentiality, required breaches to confidentiality, and how the assessment will aid in treatment planning were often sufficient. While the expected duration of the assessment had always been presented, additional information was added in order to address the question “How long is this measure?”: “The longest measure is 567 questions.” We found that by stating this information explicitly to the Veterans at the beginning of the assessment fewer Veterans voiced complaints.

While concerns regarding service connected disability benefits and the possible impact on assessment and treatment are outside the scope of the current article, time spent explaining the use of assessment results (e.g., treatment planning) and the importance of being honest in order to create a treatment plan that will benefit each Veteran has been very helpful. Additional information explaining the differences between the subdivisions of the Department of Veterans Affairs (*i.e.*, Veterans Health Administration and Veterans Benefits Administration) has also been helpful. On occasion, we have worked with Veterans whose assessment results and psychosocial interview indicated that they do not meet criteria for PTSD. For example, one Veteran’s results indicated that he met criteria for Generalized Anxiety Disorder rather than PTSD. The individual therapist was then able to share the results with the Veteran and make appropriate treatment recommendations. This Veteran initially expressed disappointment with the results; however, by the end of the feedback session the Veteran reported feeling hopeful and understood. Certainly not all Veterans respond well to feedback; however, it has been our experience that the vast majority of Veterans receive even difficult assessment feedback appropriately and find it useful.

Finally, some Veterans expressed frustration regarding having completed the same or similar measures in the past. When we explored this concern, we found that much of this frustration arose from completing assessment measures in the military and in the VA and never receiving feedback on the results. Reminding Veterans that their results will be shared with them by their individual therapist often sufficiently addressed this concern.

Even after efforts to inform clinicians of the benefits of the assessment clinic and to provide information on each of the measures, it became clear that ongoing training was needed. A train-implement-train model, with spaced trainings, has been shown to enhance the process of implementation and prevent drift [11]. One minimally invasive way that we found useful to communicate the necessary information was to incorporate review of assessments into team meetings on a periodic basis. Since difficult cases were often brought up for discussion in team meetings, one or more clinicians would retrieve the Veteran’s assessment results to offer additional information to assist with treatment planning. Often a simple review of the assessment measure and then presentation of assessment results helped remind clinicians of the content of each measure, as well as the benefits that assessment can offer.

Given the need for timely assessments in order to examine treatment change, it quickly became apparent that a tracking system was needed. Within our residential program, an Excel file was used to monitor upcoming admissions and discharges. This existing system provided an avenue for scheduling assessments. Admitting Veterans were simply scheduled for the next available assessment clinic slot, and Veterans completing treatment were scheduled for the assessment clinic just prior to their discharge date. It then became necessary to add an additional time slot on the master list for those Veterans transitioning to a different treatment modality or level of care (e.g., Veterans who complete a coping skills phase of treatment and transition to trauma-focused treatments such as Cognitive Processing Therapy or Prolonged Exposure). The provider managing the assessment clinic assumed responsibility for adding this information to the Excel file as these transitions are discussed among clinicians during team meetings.

With so many assessments, the need to ensure that feedback was consistently provided to Veterans also became apparent. In the initial stages of implementation, some clinicians reported forgetting to provide feedback on the assessment results. In order to address this, a procedure was created in which the student who enters the assessment results into the medical record adds the patient’s primary therapist as a cosigner. In this way, a reminder is created indicating that feedback should be incorporated into the next scheduled individual appointment. As with any new process, we found that once clinicians got into the routine of providing assessment feedback to Veterans this concern became less of an issue.

Certainly there may be providers that intentionally forget to provide assessment feedback or simply do not see the value in assessment. In these cases it is important to have administrative support for this process, as supervisors may be able to intervene. Additionally, part of the role of the implementation champion is to meet with these providers in order to specifically address concerns or questions. In our case, these meetings with supervisors and the implementation champion, as well as team meetings in which assessment results were reviewed helped with this process. We actually found that positive comments made by other providers who already knew or quickly learned the value and importance of assessment, positively influenced the behavior and viewpoint of these holdout clinicians. We also found that soliciting suggestions from the unconvinced providers for ways to improve the assessment process (e.g., “I don’t find much benefit from Measure X. I’m more interested in Y. Can we find a measure of that?”) has been helpful in promoting buy-in.

Finally, training other clinicians to monitor the assessment clinic was identified as a new goal to support the clinic. Although one clinician is appointed to oversee the assessment process, a contingency plan needs to be in place for sustainability. In cases where the clinician is ill, on vacation, or has conflicting obligations, other clinicians must be able to step in to keep the process running smoothly. Therefore, a written set of procedures was developed detailing the assessment process. Other clinicians were invited to observe an assessment session to become familiar with the processes. And, back-up assessment materials were printed and filed in the assessment clinic room for ease of access in cases where somebody needed to substitute on short notice.

Watching for opportunities is also an important part of the *Study-Act* portion of the cycle. For example, we already knew that the location of the clinic was not ideal by studying the timing of administrations and overflow on some administration dates. Adaptations had been made in the number of days the clinic was offered to try to address this. However, when a larger room became available in the medical center due to another clinic relocating to new spaces, the assessment clinic was reassigned to this room because it could accommodate more computers and Veterans. The larger testing space allowed for a greater number of Veterans to receive testing at the same time, which also shortened the wait time for assessment results and feedback as well as reducing the man hours necessary to administer the testing sessions.

## 12. Responses from Veterans and Clinicians

Given the goals of the assessment clinic in the PTSD-RRTP, timely feedback to the Veteran is viewed as an important step. During the feedback session, the Veterans are also given an opportunity to ask questions and provide their opinion and perspective on the assessment results. Overall, feedback from Veterans has been positive and many note that they learned valuable information that clarified their treatment and recovery planning. Some Veterans reported initial apprehension about the psychology assessment stating, “You’re going to tell me I’m crazy,” but later they indicated that receiving testing feedback actually helped to normalize and accurately label their experiences. Many Veterans have commented on the accuracy of the testing results, reflected in statements such as “That test got me so well. It took what was on the inside and put it on the outside.”

Two main criticisms from Veterans about the assessment clinic are the length of time needed to complete initial testing and anxiety regarding using a computer to answer questions. As a result of this feedback, testing administration now includes a brief orientation to the computer mouse and keyboard and a more detailed description of how long the measures will take. These steps added during the first few minutes of the assessment clinic appear to have positive effects.

One common concern expressed by providers at the inception of the implementation process was related to providing difficult assessment feedback to Veterans, and, in particular, feedback regarding invalid assessments. Certainly this concern is warranted because in a few cases Veterans have not responded well to assessment feedback. Typically, this has occurred with invalid results in which the Veteran has over-endorsed or exaggerated symptoms. In these select cases, the Veterans have become agitated, using profanity or calling the assessment results “pointless”. Sometimes even the provider has been made the target of the Veteran’s frustration, with Veterans saying “You’re telling me I’m lying” or “You don’t know what you’re talking about.” While this type of interaction is unpleasant and something providers wish to avoid, it is certainly part and parcel of our work as clinicians. For this reason, extensive discussions of how to successfully provide feedback regarding invalid results was conducted with providers prior to implementation of the assessment clinic. In these cases, knowing and feeling confident in the assessment measures and the reliability and validity of the measures is immensely helpful. Clinicians were taught to aid the Veteran by restating how the results were gathered (*i.e.*, by self-report) and therefore, the clinician can only give feedback based on how the Veteran responded: “I can only tell you what the assessment tells me and that is based on your responses.” Approaching the results in this manner allows the clinician to feel less personally attacked and helps the Veteran to see, if they are willing, that the results are a direct consequence of their response pattern. Some Veterans may request information regarding the validity of the measures used. Granted, this has only happened once with the hundreds of Veterans we have assessed; however, this Veteran was referred to the clinician who was most knowledgeable of the psychometrics of the instruments, and information was provided to the Veteran to satisfy his questions.

It is important to note that in many cases where assessment results are invalid and the Veterans have over-endorsed or exaggerated symptoms, Veterans have responded in an adaptive manner to this feedback. Many of these Veterans essentially own their responses and admit that they exaggerated or “blew things out of proportion.” In these cases, it is very helpful to use this information therapeutically. Our providers shared that they were able to work collaboratively with these Veterans to identify how this approach had been unhelpful, had been a problematic pattern across their relationships (e.g., “my family tells me the same thing”), or had been influenced by other factors (e.g., service connection, previous traumas). This allows providers the opportunity to use even invalid results to help with treatment planning.

It is also important to note that invalid results do not necessarily mean that a patient does not have a disorder. We can often reassure Veterans that they may indeed need treatment, while discussing the value of an honest and accurate portrayal of their current circumstances and symptoms in order to provide the best treatment. On a few occasions, we have found it beneficial to re-administer the instruments after having a productive discussion with a patient regarding this issue.

Feedback from individual providers has been gathered at several points since the implementation of the assessment clinic. Overall, the feedback from staff has been positive and has fallen into three main categories. First, staff members find the assessment data useful in making treatment recommendations. Second, they find the information helpful for clarifying the diagnostic picture with the Veteran. Third, the staff appreciate having objective measures of treatment progress that can be shared during feedback sessions. We consider these positive responses a reflection of the time, care, and effort we took during the implementation process.

## 13. Future Directions

As with any implementation effort, an eye on the future is always needed. Deming conceptualized the PDSA cycle as a continuous improvement effort with a feedback loop and cycles repeating as new information is gathered. In this spirit, future directions for the assessment clinic include evolution of assessment measures, program evaluation, research, and expansion. Several cycles have already been repeated, with some of the changes described below.

With all of the information collected in the assessment clinic, it became possible to aggregate the data and examine trends and treatment outcomes for program evaluation purposes. Undergraduate psychology students from nearby universities were invited to volunteer at the medical center to enter assessment results in a centralized, deidentified database. Again, the use of students minimizes the amount of time invested by clinicians, while providing valuable training opportunities for the students. The database makes information more accessible for examining characteristics of Veterans in the program (e.g., demographic information, initial symptom severity, and personality characteristics) and for programming changes. In addition, summary information can now easily be provided to clinicians, patients, hospital administrators, accrediting bodies, *etc.*

This database supported programming changes such as the addition of an ACT/DBT skills-based group. Staff had noticed an increase in associated features that often complicated treatment in incoming Veterans. The literature on complex PTSD suggests that chronic affect dysregulation, disturbances in relationships, dissociation, negative belief systems, and somatization sometimes occur in response to extreme or prolonged stressors, particularly early in an individual’s life [3,4,5]. Similarly, ongoing efforts to clarify varying presentations of PTSD have provided evidence that a small proportion of people with PTSD fit into a distinct category demarcated by high levels of dissociation [41]. The information collected in the assessment clinic supported programming changes to address these factors through the addition of an ACT/DBT skills-based group, which has been very successful in the PTSD-RRTP. Adding an instrument to assist with early detection of these associated features among individual Veterans may also be considered in the future.

Repeated measures throughout programming are included in the longitudinal database that is available for study, which offers many opportunities for contrasting outcomes and monitoring the quality of service delivery. For example, the addition of Prolonged Exposure Therapy to our treatment offerings created additional burden on the system since it is provided in an individual format while Cognitive Processing Therapy is provided in a group format. As data accumulates, we can compare outcomes to help guide decisions regarding future programming based on the effects on the Veterans’ recovery rather than on efficiency alone.

Another example of the benefit of the assessment clinic in program evaluation was demonstrated during a recent review of the BCVA residential program by an accrediting body. An up to date summary of treatment outcomes was easily available to front-line staff, administrators, and surveyors. This information was not only important for accurate review by surveyors, but it also provided staff and administrators greater accuracy and confidence when describing the benefits of the current treatment program.

As the residential treatment program evolves and changes, so too must the instruments utilized in the assessment clinic. One example of this involved the addition of mindfulness-based treatments in our residential program described above. The initial assessment measures simply did not assess the concept of mindfulness or changes that may occur related to learning and mastering mindfulness. This led to a second search, utilizing the criteria described above, to identify reliable and validated measures that could be added to the battery to assess mindfulness. The inclusion of a new instrument prompted the clinicians to re-examine the existing measures. It was important not to burden the Veterans by continuously adding additional measures and thus the amount of time needed to complete the assessment process. To this end, a review of the original battery was conducted with the knowledge that one or more of the assessment measures should be dropped to allow for inclusion of the new instrument.

The database again became a primary source of information. Rather than basing the decision solely on which tests clinicians “liked” the most, an examination of the database was conducted to determine which measures were most sensitive to change and which measures added the least amount of incremental validity. Clinicians were also polled about which measures they found most helpful and least helpful in terms of diagnosis, treatment planning, and discussions with clients. The least helpful measure, according to statistical analysis and consensus among clinicians, was dropped to make room for the desired measure. This process of choosing and eliminating measures also allowed clinicians to feel a greater sense of ownership in the overall assessment process, promoting additional buy-in.

In this manner, our assessment clinic is constantly evolving. As new measures become available or new needs are identified, we have the opportunity to consider adding better or updated measures to the battery. For example, we are considering shifting from the MMPI-2 to the MMPI-2-RF due to the shorter administration time and improved psychometric properties of the restructured clinical scales [42]. Understandably, clinicians are sometimes hesitant to switch to new measures. In order to increase acceptability, an expert from a nearby university was invited to the medical center to provide a 90-minute training on the MMPI-2-RF. This allowed the staff to increase their familiarity with the updated measure and ask questions about a possible transition.

As noted above, the current assessment process allows the opportunity to conduct research. One of the interests currently under study is the search for markers that predict treatment outcomes. Clinicians often are looking for clues that may help them determine how well a Veteran will do in a particular treatment, which Veterans may gain the most from a treatment, and contraindications for a treatment. To this end, we have included measures in the assessment process that may help us determine the answer to some of these questions. For example, Cognitive Processing Therapy, one of the treatments offered in our residential program, requires that an individual learn to challenge and modify unrealistic and maladaptive thoughts that interfere with recovery. It may be that individuals who demonstrate more flexibility in thinking fare better in this treatment. To this end, a measure of cognitive flexibility has been added to the assessment process with the hope that this clinical question can be answered and disseminated to the larger psychological community.

With the success of the assessment clinic in the PTSD-RRTP, clinicians outside the program began noticing the added benefit of assessment in terms of diagnostic clarity and treatment planning. Providers involved in the implementation process have been asked to share their knowledge and act as consultants for other programs that begin this process. The implementation of additional assessment clinics is being considered in our Psychosocial Residential Rehabilitation Treatment Program and currently taking place in our PTSD outpatient clinic. In the outpatient clinic, this process, similar to our initial assessment clinic, began with a discussion of goals. Currently, the logistics of the clinic are being considered in a manner that may be mutually beneficial to all programs involved. The possibility of sharing clinic space (e.g., computers and room), as well as combining forces to proctor the assessments has been discussed. The possibility of sharing resources can allow providers in multiple programs to obtain the desired information with minimal man hours, which increases the likelihood for sustainability.

Our overall approach to the implementation of this assessment clinic appears to be successful since we have been able to assess and utilize the data from 413 Veterans admitted to the PTSD-RRTP as of the time of this writing. In addition, the data that we have collected has been useful for program evaluation and shared throughout our medical center in Grand Rounds presentations to educate providers from other disciplines and clinics about the value of PTSD treatment. There are also a number of pilot projects underway to publish articles on various hypotheses regarding treatment in residential PTSD programs. Our hope is that the current assessment process not only improves the day-to-day operations of the program, but also serves to inform future changes to our program as well as disseminating knowledge to the larger psychological community.

## 14. Conclusions

Clinicians who seek to implement a similar assessment clinic in their own setting are unlikely to be able to follow the framework described here step-by-step since every setting has its own unique set of challenges and resources. Even within the VA system, there is a great deal of variability between stations. For example, other facilities may find that a psychology technician is available or can be hired to administer the test sessions. The use of students would then be unnecessary and alleviate a great deal of burden on the system. Another example of differences that may need to be addressed would be the case of a managed care setting. Assessment for every patient entering treatment may not be feasible in a system that does not routinely pay for such services. Therefore, reducing the assessment to a simpler form that could be accommodated in the normal course of treatment may be necessary. Clinicians could then obtain authorization for a more involved assessment when it was justified for a particular case. In fact, we are aware of another VA facility that uses this model and we considered this arrangement in the early stages of planning. Clinicians wishing to establish an assessment clinic will need to focus on the specific goals that they are hoping to accomplish, as well as the particular resources and barriers at their facility in order to help guide the process of implementation. The purpose of our detailed description was to illustrate the process of analyzing a system, organizing a change, and overcoming barriers. Through the use of a framework similar to the FOCUS-PDSA model, differences such as these will be exposed so that the process can be refined for any other system.
